# Identification of Silencing Suppressor Protein Encoded by Strawberry Mottle Virus

**DOI:** 10.3389/fpls.2022.786489

**Published:** 2022-05-31

**Authors:** Lingjiao Fan, Chengyong He, Dehang Gao, Tengfei Xu, Fei Xing, Jiaqi Yan, Binhui Zhan, Shifang Li, Hongqing Wang

**Affiliations:** ^1^Department of Fruit Science, College of Horticulture, China Agricultural University, Beijing, China; ^2^State Key Laboratory of Biology of Plant Diseases and Insect Pests, Institute of Plant Protection, Chinese Academy of Agricultural Sciences, Beijing, China

**Keywords:** strawberry mottle virus, RNA silencing suppressor, subcellular localization, conserved GW motif, symptom determinant

## Abstract

Strawberry mottle virus (SMoV) is associated with strawberry decline disease, causing losses to fruit yield and quality. In this study, using a screening system that enables detection of both local and systemic plant host (RNA silencing) defense responses, we found that Pro2Glu and P28, encoded by SMoV RNA2 genome, functioned to suppress local and systemic RNA silencing triggered by single- but not double-stranded GFP RNA. Subcellular localization assay revealed that both Pro2Glu and P28 were localized to nucleus and cytoplasm. The deletion of 11 amino acid residues at the C-terminus destabilized Pro2Glu protein, and the disruption of two conserved GW motifs deprived Pro2Glu of ability to suppress RNA silencing. Additionally, SMoV Pro2Glu and P28 enhanced the accumulation of potato virus X (PVX) in *Nicotiana benthamiana* 22 days post-infiltration, and P28 exacerbated significantly the symptoms of PVX. Collectively, these data indicate that the genome of SMoV RNA2 encodes two suppressors of RNA silencing. This is the first identification of a stramovirus suppressor of RNA silencing.

## Introduction

Plant viruses are biotrophic pathogens that need living tissue to complete their multiplication cycle, and thus they interfere and/or compete with host for a substantial amount of host resources (Pallas and Garcia, 2011; Mandadi and Scholthof, 2013). To defend against invading viruses, plants employ several layers of immune responses. Among these immune responses, RNA silencing is a conserved eukaryotic gene regulation mechanism that provides sequence-specific antiviral defense (Covey et al., 1997; Baulcombe, 2004; Ding et al., 2004; Gupta et al., 2019). Briefly, the process of RNA silencing is that double-stranded RNA (dsRNA) are cleaved by Dicer-like RNases (DCLs) into 21–24 nucleotides (nt) small interfering RNAs (siRNAs), and then the siRNAs are incorporated into an Argonaute (AGO)-containing RNA-induced silencing complex (RISC) to degrade target RNAs and/or to inhibit its translation (Pallas and Garcia, 2011). In addition, the siRNAs and target RNAs can synthesize dsRNAs in the presence of RNA-dependent RNA polymerases (RDRs), thus amplifying the antiviral silencing signal (Sijen et al., 2001; Parent et al., 2015; Gupta et al., 2019). Furthermore, the siRNAs also can act as mobile silencing signals that can trigger local silencing by moving from cell to cell *via* plasmodesmata as well as systemic silencing *via* phloem companion cells transport (Melnyk et al., 2011; Pumplin and Voinnet, 2013; Yang et al., 2018).

To counter RNA silencing, viruses encode one or more viral suppressor(s) of RNA silencing (VSR), which function in various steps of the process of RNA silencing (Li and Ding, 2006; Valli et al., 2009; Nakahara and Masuta, 2014; Csorba et al., 2015). The 2b protein of cucumber mosaic virus (CMV) and the p38 protein of turnip crinkle virus (TCV) both interact with AGO1 and inhibit AGO1-mediated gene silencing, but using different mechanisms: 2b through interaction with the PAZ domain and p38 through GW motifs that serve as an Ago “hook” (Danielson and Pezacki, 2013). Concurrently, 2b also binds small RNAs generated upon viral infection by DCLs, and this pathway was considered to be the most relevant in determining the suppressor functionality (González et al., 2010; Danielson and Pezacki, 2013). The P19 protein of tombusviruses, having a high affinity to duplexes 20–22 nucleotides long, functions to sequester small RNA duplexes (Cheng et al., 2011). In addition, P19 also suppresses RNA silencing by inducing the expression of a host miRNA (miR168) that reduces concentration of AGO1, thereby inhibiting RISC formation (Varallyay et al., 2010; Danielson and Pezacki, 2013).

Strawberry mottle virus is the most common virus of strawberry and occurs naturally in the genus *Fragaria* worldwide (Freeman and Mellor, 1962). It is one of the viruses found in association with strawberry decline disease. Its severe strains may reduce vigor and yield by up to 30% in strawberry cultivars (Martin and Tzanetakis, 2006). SMoV has been classified into subgenus *Stramovirus* within the genus *Sadwavirus*, family *Secoviridae* (Sanfaçon et al., 2020). SMoV has two positive-sense RNA genome segments, whereby RNA1 encodes a large polyprotein (about 215 kDa), referred to as P1. The P1 is cleaved in cis by 3C-like protease (3CL-Pro) at five sites to release mature proteins, including a putative RNA helicase (Hel), a 3CL-Pro (Pro), a viral genome-linked protein (Vpg), and an RNA-dependent RNA polymerase (RdRp) at its C-terminus, and two unknown proteins X1 and X2 at its N-terminus. The RNA2 encodes a polyprotein of about 190 kDa, referred to as P2. The P2 is cleaved in cis by glutamic protease (Pro2Glu) to form a 28 kDa unknown function protein (P28) and a Pro2Glu (40 kDa) at its C-terminus; additionally, a movement protein (MP) and a coat protein (CP) at its N-terminus are released with the help of 3CL-Pro (Bhagwat et al., 2016; Mann et al., 2017, 2019). Although the cleavage sites within the polyproteins have been experimentally defined largely, some proteins function is still ill-defined and even unknown.

For the family *Secoviridae*, several VSRs have been identified previously, including the R78 polyprotein of the *waikavirus* maize chlorotic dwarf virus and bellflower vein chlorosis virus (Stewart et al., 2017); the coat protein of the *nepovirus* tomato ringspot virus (Karran and Sanfaçon, 2014); the VP53, VP37 and large capsid protein of the *fabavirus* broad bean wilt virus 2 (Kong et al., 2014); the Vp20 of the *cheravirus* apple latent spherical virus (Yaegashi et al., 2007); and the small coat protein (SCP) of the *comovirus* cowpea mosaic virus (CPMV; Liu et al., 2004). In the present study, we found two suppressors, Pro2Glu and P28, encoded by SMoV RNA2 genome using a screening system that enables detection of both local and systemic plant host RNA silencing. Under the similar experimental conditions, we showed that Pro2Glu and P28 were unable to suppress local RNA silencing triggered by double-stranded GFP RNA. We also reported that Pro2Glu and P28 proteins were localized to nucleus and cytoplasm. Furthermore, G_5_W_6_ and G_116_W_117_ motifs of Pro2Glu were indispensable to suppressor activity. Additionally, Pro2Glu and P28 enhanced the accumulation of potato virus X (PVX) in *Nicotiana benthamiana* leaves, and P28 aggravated the symptoms of PVX.

## Materials and Methods

### Plant Materials

Wild type (WT) *N. benthamiana* plants, green fluorescent protein (GFP) transgenic (line 16c) *N. benthamiana* plants and red fluorescent protein (RFP) transgenic *N. benthamiana* plants were used in the present study. They were grown from seeds in an insect-free growth room at 25°C under a 16 h light and 8 h dark photoperiod. The third and fourth leaves from the top were used for infiltration and inoculation experiments when the plants were about 4 weeks old.

### Plasmid Construction

Ten SMoV protein coding regions, including X1, X2, Hel, Pro, Vpg, RdRp encoded by RNA1, MP, CP, Pro2Glu, and P28 encoded by RNA2, were individually amplified by polymerase chain reaction (PCR) from the SMoV Chinese isolate DGHY21 (GenBank accession no. MT070753) according to the method described previously (Fan et al., 2021). Specific primers used in this study were listed in Supplementary Table S1 (containing suitable restriction sites). The PCR products were cloned individually into the pTOPO-Blunt vector (Aidlab, Beijing, China) to generate pTOPO-X1, pTOPO-X2, pTOPO-Hel, pTOPO-Pro, pTOPO-Vpg, pTOPO-RdRp, pTOPO-MP, pTOPO-CP, pTOPO-Pro2Glu, and pTOPO-P28, which were individually digested with specific enzymes for subsequent cloning.

To screen the VSRs, each protein-coding region was subcloned into the pCHF3 vector (Cai et al., 2007) under the control of a 35S promoter and NOS terminator to generate pCHF3-X1, pCHF3-X2, pCHF3-Hel, pCHF3-Pro, pCHF3-Vpg, pCHF3-RdRp, pCHF3-MP, pCHF3-CP, pCHF3-Pro2Glu, and pCHF3-P28. The resulting recombinant pCHF3 constructs were chemically transformed into the *Agrobacterium tumefaciens* strain EHA105.

For the subcellular localization analysis, the full-length fragments of SMoV Pro2Glu and P28 were inserted into, respectively, the *Sma* I/*Kpn* I and *Apa* I/*Kpn* I sites of pCAMBIA1300-GFP vector to produce C1300-Pro2Glu and C1300-P28 that contained GFP fusion protein. The resulting recombinant pCAMBIA1300-GFP constructs were chemically transformed into the *A. tumefaciens* strain GV3101.

For the pathogenicity test, the full-length coding regions of Pro2Glu and P28 were subcloned into the PVX-containing pGR106 vector (a kind gift from David C. Baulcombe, University of Cambridge, Cambridge, United Kingdom) between the *Cla* I and *Sal* I restriction sites to produce PVX-Pro2Glu and PVX-P28. The *A. tumefaciens* strain GV3101 (pJIC SA_Rep) was transformed with the two plasmids.

To generate the Pro2Glu and P28 derivatives, the Glu^dm329–339aa^ mutant with the 329GVSNRKKHRRG339 motif deleted was amplified by PCR from the plasmid pTOPO-Pro2Glu with primers Glu^dm329–339aa^-F/R. The resulting Glu^dm329–339aa^ was inserted into the *Bam*H I/*Pst* I sites of pCHF3 or *Sma* I/*Kpn* I sites of pCAMBIA1300-GFP to produce pCHF3-Glu^dm329–339aa^ or C1300-Glu^dm329–339aa^, respectively. The GW motifs of Pro2Glu and P28 were disrupted through W_6_A (m4Glu), W_117_A (m1Glu), W_172_A (m2Glu), and W_191_A (m3P28) site-directed mutagenesis using gene synthesis by Sangon Biotech (Shanghai, China) Co., Ltd. The resulting mutants were inserted into the *Bam*H I/*Pst* I sites of pCHF3 to produce pCHF3-m4Glu, pCHF3-m1Glu, pCHF3-m2Glu, and pCHF3-m3P28.

### *Agrobacterium* Infiltration and GFP Imaging

*Agrobacterium*-mediated transient gene expression was carried out based on developed protocols (Hamilton et al., 2002). *Agrobacterium* cultures were pelleted and resuspended to an optical density OD_600_ = 1.0 in a solution containing 10 mM MgCl_2_, 10 mM MES (pH 5.8), and 200 μM acetosyringone, and were incubated at room temperature in dark for 3 h before infiltration. For co-infiltration, equal volumes of *Agrobacterium* cultures harboring 35S-GFP (a 35S promoter-driven construct expressing sense GFP, a kind gift from Prof. Xiuling Yang, Chinese Academy of Agricultural Sciences, Beijing, China) and the tested constructs were mixed, followed by infiltration into fully expanded leaves of 4-week-old 16c or WT *N. benthamiana* plants. A construct pCHF3-P19 to express tomato bushy stunt virus (TBSV) P19 was used as a positive control, and an empty vector pCHF3 was used as a negative control. GFP fluorescence in infiltrated or systemic leaves was monitored under a handheld long-wavelength UV lamp (Black-Ray Model B-100A, San Gabriel, CA, United States) and was photographed with a Canon EOS-M digital camera featuring a 55 mm yellow filter.

### Northern Blot Analysis

Total RNA was extracted from *Agrobacterium*-infiltrated leaf patches of *N. benthamiana* line 16c or *N. benthamiana* WT plants by using an E.Z.N.A.^®^ Plant RNA Kit (Omega Bio-tek, Norcross, GA, United States) following the manufacturer’s instructions. Then, 2.5 μg of total RNA was separated on 1.5% formaldehyde-agarose gels and was transferred to Hybond N^+^ membranes as instructed (GE Healthcare, Buckinghamshire, United Kingdom). The membrane was hybridized with a digoxigenin-labeled GFP probe, which was made using a PCR DIG probe synthesis kit, and was detected using a detection starter kit II according to the manufacturer’s instructions (Roche Diagnostic, Basel, Switzerland). GelStain (Transgen, Beijing, China) staining was used to visualize the loading controls for the mRNA.

For siRNA blot assay, total RNA were extracted from infiltrated leaves with Trizol reagent (Transgen, Beijing, China) as recommended by the manufacturer. Low-molecular-mass RNAs were enriched from total RNA by adding equal volume of 10 M lithium chloride and incubating 4 h on ice. The RNA was centrifuged at 13,000 rpm for 15 min. The supernatant was precipitated at −20°C for at least 2 h after adding three volumes of ethanol. The low molecular weight (LMW) RNAs were collected by centrifugation for 15 min at 13,000 rpm. The enriched small RNAs were fractionated on a 15% denaturing polyacrylamide (19:1) gels containing 7 M urea in 0.5 × TBE buffer, then transferred to Hybond N^+^ membranes by electroblotting at 20 V for 60 min, and chemically cross-linked *via* 1-ethyl-3-(3-dimethylaminopropyl) carbodiimide (EDC) as described previously (Pall and Hamilton, 2008). The membrane was hybridized with the digoxigenin-labeled GFP probe which was hydrolyzed into about 50 nt long RNA pieces by treatment with sodium carbonate buffer as described by Dalmay et al. (2000) at 40°C overnight, subsequently, washed at 50°C in 2 × SSC (0.3 M and 0.03 M sodium citrate) and 0.2% sodium dodecyl sulfate.

### Western Blot Analysis

Total soluble proteins were extracted from *Agrobacterium*-infiltrated leaf patches of *N. benthamiana* WT or *N. benthamiana* line 16c plants by using a Plant Total Protein Lysis Buffer (Sangon Biotech, Shanghai, China). The protein samples were separated by SDS-PAGE. The anti-GFP mouse monoclonal antibody (TransGen Biotech, Beijing, China) was used at 1:5,000 dilution. Western blots were visualized with a goat anti-mouse IgG (H + L) HRP conjugate (TransGen Biotech, Beijing, China) and a chemiluminescence detection system (Tianneng, Shanghai, China). Coomassie blue staining of the large subunit of Rubisco served as loading controls for the Western blot assay.

### Protein Interaction Assay

The Y2H experiment was performed using Super Yeast Transformation Kit (Coolaber, Beijing, China) according to the manufacturers. Plasmids of the bait and prey pairs were co-transformed into yeast cells and cultured at 30°C for 72 h on double dropout (−Leu/−Trp) or quadruple dropout (−Ade/-His/−Leu/−Trp) synthetic dextrose (SD) medium, respectively.

### Quantitative RT-PCR Analysis

Total RNA was extracted from *N. benthamiana* systemic leaves infected by PVX, PVX-Pro2Glu and PVX-P28 at 22 dpi. Reverse transcription was conducted according to the published method (Fan et al., 2021). The RT-qPCR reactions in the 20 μl volume containing 1 μl cDNA as template were run using GoTaq qPCR Master Mix (Promega, Madison, United States) according to the instructions supplied by the manufacturer on a MyGo Pro real-time PCR instrument (IT-IS Life Science Ltd., Republic of Ireland). Primers G-35 and G-36 were used for determining PVX genomic RNA accumulation as described elsewhere (Gupta et al., 2018). *PP2A* was chosen as the internal control for normalization of virus accumulation in *N. benthamiana* plants. The 2^-ΔΔCT^ method was used to analyze the RT-qPCR expression data according to Bustin et al. (2009). GraphPad (GraphPad Software Inc., San Diego, CA, USA) was used to analyze the experimental data by one-way ANOVA, and multiple comparisons were done using Tukey’s test (*p* < 0.05).

### Laser-Scanning Confocal Microscopy

Imaging of fluorescent proteins was conducted using a confocal microscopy (LSM880; Carl Zeiss, Jena, Germany) at 60 h post-infiltration. To confirm the nuclei of the leaf epidermal cells, RFP transgenic *N. benthamiana* leaves were infiltrated with *Agrobacterium* cultures harboring the C1300-Pro2Glu or C1300-Glu^dm329–339aa^ construct. For GFP, excitation was performed at 488 nm, and emission was recorded at 500 to 530 nm. For RFP, excitation was done at 561 nm, and emission was recorded at 590 to 630 nm.

### Pathogenicity Enhancement Assay

*Agrobacterium* cultures harboring the PVX-Pro2Glu and PVX-P28 vectors were diluted to approximately OD_600_ = 0.8 prior to infiltration into leaves of *N. benthamiana* plants. The infiltrated plants were examined for virus-like symptoms and photographed at various time-points after infiltration.

## Results

### Pro2Glu and P28 Both Suppress Local RNA Silencing Triggered by Single- but Not Double-Stranded GFP RNA

**Figure 1:**
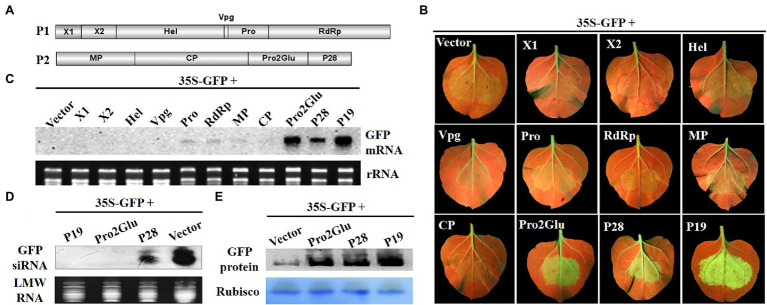
The identification of SMoV silencing suppressor. **(A)** The genomic structure of SMoV. **(B)** The observation of GFP fluorescence in 16c *Nicotiana benthamiana* leaves co-infiltrated by 35S-GFP and pCHF3 vectors expressing individual proteins of SMoV at 5 days post-infiltration (dpi). The plants infiltrated with 35S-GFP and pCHF3 empty vector or pCHF3-P19 were used as negative or positive controls, respectively. **(C)** The analysis of GFP mRNA in infiltrated leaf patches by Northern blot at 5 dpi. DIG-labeled GFP-specific probe was used to detect the GFP mRNA. GelStain (Transgen, Beijing, China) staining was used to visualize the loading controls for the mRNA. The same below. **(D)** Northern blot analysis of low molecular weight (LMW) RNA from infiltrated leaf regions at 5 dpi for GFP-specific siRNA accumulation. DIG-labeled GFP-specific probe after hydrolyzing in sodium carbonate buffer was used to detect the GFP siRNA. GelStain staining was used to visualize the loading controls for the LMW RNA. The same below. **(E)** Western blot analysis of GFP protein in infiltrated leaf regions at 5 dpi using anti-GFP monoclonal antibody. Coomassie blue staining of the large subunit of Rubisco served as loading controls for the Western blot assay. The same below.

To identify VSRs of SMoV, a classical GFP-based two-component *Agrobacterium* infiltration assay was used to screen potential VSRs. *Agrobacterium* containing 35S-GFP and plasmids expressing individual proteins (X1, X2, Hel, Vpg, Pro, RdRp, MP, CP, Pro2Glu, and P28) of SMoV (Figure 1A) were mixed and co-infiltrated into fully expanded leaves of *N. benthamiana* line 16c. Three days post infiltration (dpi), GFP fluorescence was evident in all *Agrobacterium*-infiltrated leaf patches. By 5 dpi, as a consequence of GFP local silencing, GFP fluorescence became weak in leaves co-infiltrated with 35S-GFP and the empty pCHF3 vector and the tested recombinant pCHF3 vectors expressing X1, X2, Hel, Pro, Vpg, RdRp, MP, and CP. However, GFP fluorescence remained strong in leaves co-infiltrated with 35S-GFP and pCHF3-Pro2Glu or pCHF3-P28, resembling the positive control for silencing suppression (pCHF3-P19) (Figure 1B).

The northern blot analysis was carried out using total RNAs (for GFP mRNA) or LMW RNAs (for GFP siRNA) extracted from patches of co-infiltrated leaves. Consistent with the observed GFP fluorescence, GFP mRNA accumulated to higher levels in leaves co-infiltrated with 35S-GFP and pCHF3-P19, pCHF3-Pro2Glu or pCHF3-P28 compared to empty vector (Figure 1C), however, GFP siRNA accumulated at significantly reduced levels in plants expressing P28 and was not detected in P19 and Pro2Glu expressing plants (Figure 1D). Western blot also showed the accumulation of GFP proteins was higher in the leaves co-infiltrated with Pro2Glu + GFP, P28 + GFP or P19 + GFP than in those co-infiltrated with pCHF3 + GFP (Figure 1E). These results indicate that Pro2Glu and P28 encoded by SMoV RNA2 are capable of suppressing local RNA silencing triggered by single-stranded GFP.

**Figure 2:**
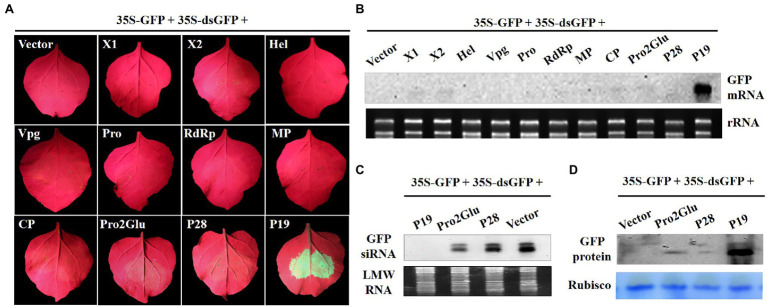
Pro2Glu and P28 failed to suppress local silencing triggered by dsGFP. **(A)** The observation of GFP fluorescence in wild-type *N. benthamiana* leaves co-infiltrated by 35S-GFP, 35S-dsGFP, and pCHF3 vectors expressing individual proteins of SMoV at 3 dpi. The plants co-infiltrated by 35S-GFP, 35S-dsGFP, and pCHF3 empty vector or pCHF3-P19 were designated as negative or positive controls, respectively. **(B)** The analysis of GFP mRNA in infiltrated leaf patches by Northern blot at 3 dpi. **(C)** Northern blot analysis of GFP-specific siRNA in infiltrated leaf regions at 3 dpi. **(D)** Western blot analysis of GFP protein in infiltrated leaf regions at 3 dpi using anti-GFP monoclonal antibody.

To explore whether Pro2Glu and P28 could suppress dsRNA-induced RNA silencing, *Agrobacterium* suspension containing 35S-GFP, 35S-dsGFP (a construct expressing an inverted repeat sequence of GFP, a kind gift from prof. Xiuling Yang, Chinese Academy of Agricultural Sciences, Beijing, China) and plasmids expressing individual proteins X1, X2, Hel, Vpg, Pro, RdRp, MP, CP, Pro2Glu, and P28 were inoculated into fully expanded leaves of *N. benthamiana*. At 3 dpi, only the leaf patches co-inoculated by 35S-GFP, 35S-dsGFP and pCHF3-P19 showed strong GFP fluorescence, whereas leaf patches co-inoculated by 35S-GFP, 35S-dsGFP and empty vector, Pro2Glu, P28 or other eight proteins all showed faded GFP fluorescence (Figure 2A). The Northern blot analysis revealed that the leaves co-inoculated with 35S-GFP, 35S-dsGFP and pCHF3-P19 accumulated high levels of GFP mRNA, while GFP siRNA was not detected. Conversely, in other treatments, GFP mRNA remained undetected (Figure 2B) and high levels of GFP siRNA were detected (Figure 2C). Correspondingly, the western blot analysis revealed that the level of GFP protein expression was stronger in leaf patches co-infiltrated by pCHF3-P19 than in other co-infiltrated treatments (Figure 2D). To confirm this observation, we conducted the assay in a leaf of *N. benthamiana*. At 3 dpi, the same results were observed (Supplementary Figure 1). These data demonstrate that Pro2Glu and P28 failed to suppress dsGFP-induced RNA silencing.

### Pro2Glu and P28 Inhibit Activation of Systemic Host RNA Silencing-Based Defense Responses

**Figure 3:**
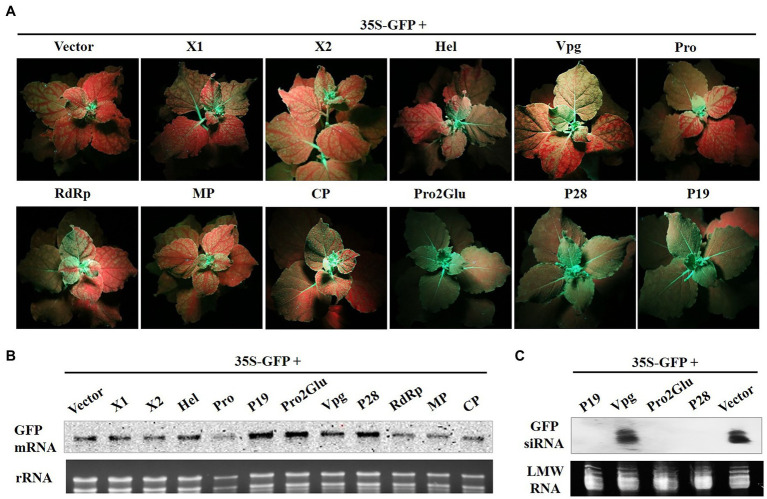
Pro2Glu and P28 inhibited the systemic silencing of GFP in *N. benthamiana* line 16c plants. **(A)** *N. benthamiana* 16c plants infiltrated with 35S-GFP and recombinant plasmids were photographed under UV light at 28 dpi. **(B)** The analysis of GFP mRNA in upper young leaves by Northern blot at 28 dpi. **(C)** The analysis of GFP-specific siRNAs in upper young leaves by Northern blot at 28 dpi.

To determine whether Pro2Glu and P28 could inhibit activation of systemic host RNA silencing based defense responses, we agroinfiltrated two fully expanded leaves of *N. benthamiana* 16c line with 35S-GFP and empty vector, pCHF3-Pro2Glu, pCHF3-P28 or pCHF3-P19. GFP fluorescence was monitored in the newly emerging leaves of infiltrated plants. The plants with major and minor veins of upper young leaves turning red were assumed to be silenced systemically. At 28 dpi, 95% of plants co-infiltrated with 35S-GFP and empty vector showed systemic silencing, but only 32.4 and 35.3% of plants co-infiltrated with, respectively, 35S-GFP plus pCHF3-Pro2Glu or pCHF3-P28 exhibited systemic silencing (Figure 3A and Supplementary Table 2). The GFP mRNA and siRNA level of plants upper young leaves were analyzed by Northern blot. The results revealed high accumulation of GFP mRNA and negligible accumulation of GFP siRNA in patches co-infiltrated with 35S-GFP plus pCHF3-Pro2Glu, pCHF3-P28 or pCHF3-P19 (Figures 3B,C). These data demonstrate that Pro2Glu and P28 effectively inhibit activation of systemic host RNA silencing-based defense responses.

Some VSRs, such as apple chlorotic leaf spot virus (ACLSV) P50, suppress systemic host RNA silencing without interfering with local silencing. To find out whether SMoV also encodes other suppressor that is unable to suppress local RNA silencing but inhibits systemic RNA silencing, agroinfiltration containing 35S-GFP and the other recombinant plasmids (pCHF3-X1, -X2, -Hel, -Vpg, -Pro, -RdRp, -MP or -CP) into fully expanded leaves of *N. benthamiana* 16c line was done. At 28 dpi, red fluorescence was observed in newly emerging leaves in every treatment with 35S-GFP and pCHF3-X1, -X2, -Hel, -Vpg, -Pro, or –MP. More than 90% of infiltrated plants showed red fluorescence in the other two treatments with 35S-GFP and pCHF3-RdRp or pCHF3-CP (Figure 3A and Supplementary Table 2). The GFP mRNA level of plants upper young leaves was analyzed by Northern blot. The results were consistent with the observed GFP fluorescence (Figure 3B). Taken together, these data indicate that X1, X2, Hel, Vpg, Pro, RdRp, MP or CP of SMoV do not efficiently inhibit systemic silencing of GFP.

### Deletion of 11 Amino Acids at the C-Terminus Destabilizes Pro2Glu Protein

To determine the subcellular localizations of Pro2Glu and P28, we generated GFP-fused constructs C1300-Pro2Glu and C1300-P28, and individually infiltrated them into *N. benthamiana* leaves. At 60 h post-infiltration, the control leaves expressing GFP showed that GFP protein was localized in the nucleus and cytoplasm. Similarly, Pro2Glu and P28 proteins were observed in the nucleus and cytoplasm (Supplementary Figure 2).

**Figure 4:**
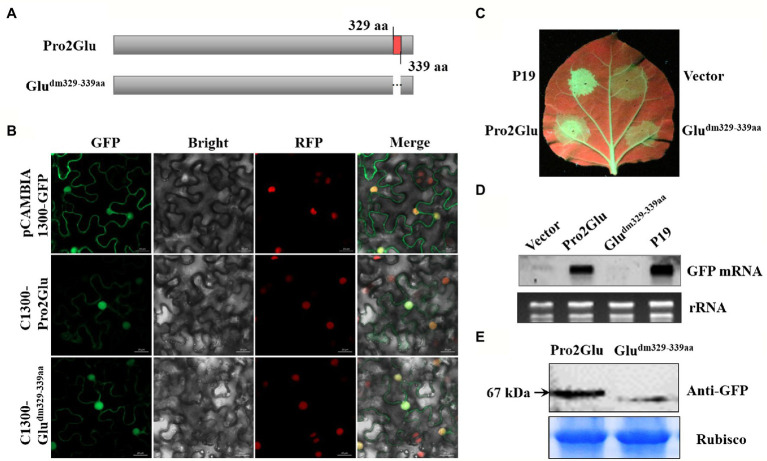
The effect of deletion of 11 amino acids at the C-terminus of Pro2Glu on the subcellular localization and suppressor activity of Pro2Glu. **(A)** Schematic representation of Pro2Glu and its deletion mutant. **(B)** Subcellular localization of Pro2Glu and Glu^dm329–339aa^ variant in leaf epidermal cells of red fluorescent protein transgenic *N. benthamiana. Agrobacterium* cultures containing pCAMBIA1300-GFP, C1300-Pro2Glu or C1300-Glu^dm329–339aa^ was infiltrated into leaves of *N. benthamiana*, respectively. Imaging of fluorescent proteins was conducted using a confocal microscopy at 60 h post-infiltrated. **(C)** The observation of GFP fluorescence in leaves of *N. benthamiana* 16c infiltrated with a mixture of *Agrobacterium* cultures containing 35S-GFP and pCHF3-Pro2Glu (bottom left of leaf) or pCHF3-Glu^dm329–339aa^ (bottom right of leaf) at 4 dpi. Expression of 35S-GFP with pCHF3-P19 (upper left of leaf) or pCHF3 vector (upper right of leaf) served as positive or negative controls, respectively. **(D)** The analysis of GFP mRNA in infiltrated leaf patches by Northern blot at 4 dpi. **(E)** Western blot analysis of total proteins from *N. benthamiana* leaf patches infiltrated with GFP-fused constructs C1300-Pro2Glu and C1300-Glu^dm329–339^ at 3 dpi using anti-GFP antibody.

Some studies reported that nuclear localization signal (NLS) is required for VSR suppressor activity (Wang et al., 2004; González et al., 2010; Yang et al., 2018). To explore which regions within Pro2Glu and P28 are crucial for their suppressor activity, we analyzed the SMoV Pro2Glu and P28 sequences using cNLS Mapper (Kosugi et al., 2009), and found that the GVSNRKKHRRG motif between amino acids 329 and 339 might be the NLS of the Pro2Glu protein, but the available NLS motif was not predicted in the P28 sequence. Hence, we deleted the 329GVSNRKKHRRG339 motif from Pro2Glu and constructed a cassette (Figure 4A; C1300-Glu^dm329–339aa^) to clarify the subcellular localization of the Pro2Glu mutant variant. Parallel with the wild-type Pro2Glu, the fluorescence of C1300-Glu^dm329–339aa^ was generally distributed in the nucleus and cytoplasm (Figure 4B). We further confirmed the nuclear localization of Pro2Glu and Glu^dm329–339aa^ in RFP transgenic *N. benthamiana* plants (Figure 4B), demonstrating that the region covering aa 329–339 is not required for nuclear localization of Pro2Glu protein.

Furthermore, Glu^dm329–339aa^ was cloned into pCHF3 to evaluate its ability to suppress RNA silencing. Fully expanded leaves of *N. benthamiana* 16c line were co-infiltrated with 35S-GFP and pCHF3-P19, pCHF3 empty vector, pCHF3-Glu^dm329-339aa^ or pCHF3-Pro2Glu, and monitored under UV light. At 4 dpi, strong GFP fluorescence was observed in the leaf patches co-infiltrated with 35S-GFP and pCHF3-Pro2Glu or pCHF3-P19. However, GFP fluorescence was weak in the leaf patches co-infiltrated with 35S-GFP and pCHF3-Glu^dm329-339aa^ or empty vector (Figure 4C). This observation was confirmed by the Northern blot analysis that examined the relative GFP mRNA level extracted from the corresponding leaf patches (Figure 4D). To determine whether the 329GVSNRKKHRRG339 motif deletion destabilized Pro2Glu protein. Western blot analysis was performed to detect the GFP fusion proteins using anti-GFP antibody. Detection of the expected protein sizes revealed that the deletion of the 329GVSNRKKHRRG339 motif significantly decreased the level of the Pro2Glu protein (Figure 4E). Taken together, these results demonstrate that the 329GVSNRKKHRRG339 motif from Pro2Glu is not necessary for Pro2Glu nucleus localization. Since the 11 amino acids deletion makes the Pro2Glu unstable, the role of this motif in RNA silencing remains inconclusive.

### Two GW Motifs of Pro2Glu Interfere With Suppressor Activity

**Figure 5:**
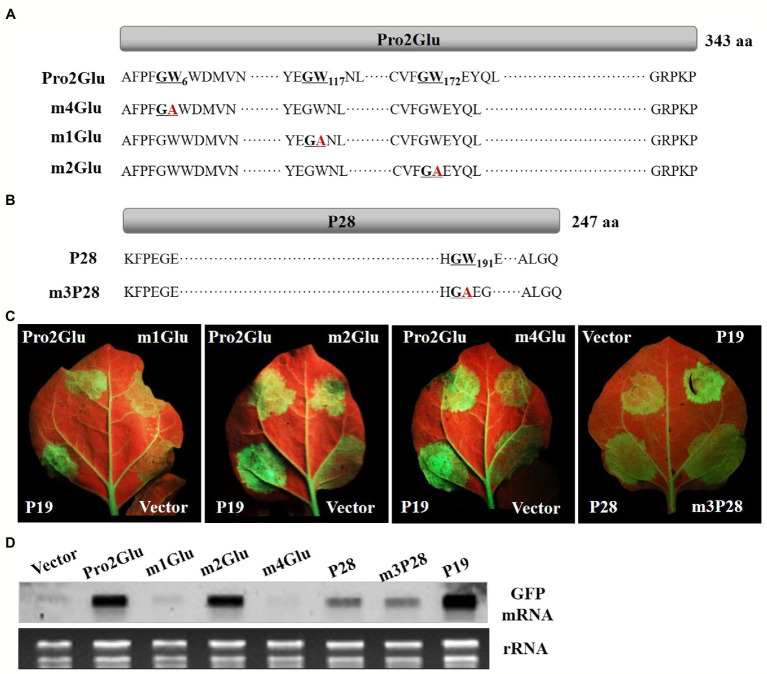
The effect of GW motifs on the suppressor activity of Pro2Glu and P28. **(A)** Schematic representation of Pro2Glu and the mutation sites of its mutants. **(B)** Schematic representation of P28 and the mutation site of its mutant. **(C)** The observation of GFP fluorescence in leaves of *N. benthamiana* 16c infiltrated with *Agrobacterium* cultures containing 35S-GFP, plus pCHF3-Pro2Glu, pCHF3-P28 or their derivatives at 4 dpi. Expression of 35S-GFP with pCHF3-P19 or pCHF3 vector served as positive or negative controls, respectively. **(D)** The analysis of GFP mRNA in infiltrated leaf patches by Northern blot at 4 dpi. The each RNA of lane of Vector, Pro2Glu and P19 is the same as used in Figure 4D.

The GW motif has been considered to be involved in direct interaction with AGO proteins to inhibit AGO-mediated gene silencing (El-Shami et al., 2007; Lian et al., 2009; Gupta et al., 2019). We analyzed the Pro2Glu and P28 sequences, and identified three conserved GW motifs in Pro2Glu of all available SMoV isolates (Supplementary Figures 3A–C) and a conserved GW motif in P28 of Chinese SMoV isolates (Supplementary Figure 3D). These GW motifs were disrupted through WA site-directed mutagenesis (Figures 5A,B). The resulting mutants were confirmed by Sanger sequencing (Supplementary Figure 4). Agroinfiltration of 35S-GFP along with pCHF3-m1Glu or pCHF3-m4Glu into leaves of *N. benthamiana* 16c line showed weak green fluorescence under UV light at 4 dpi compared to leaves co-infiltrated with wild-type pCHF3-Pro2Glu. Concurrently, leaves agroinfiltrated with pCHF3-m2Glu or pCHF3-m3P28 showed green fluorescence similar to wild-type pCHF3-Pro2Glu or pCHF3-P28, respectively (Figure 5C).

GFP expression was tested by Northern blot hybridization of total RNA extracted from infiltrated regions at 4 dpi. The results showed that GFP mRNA accumulated at lower levels in pCHF3-m1Glu- or pCHF3-m4Glu-infiltrated leaf patches compared with wild-type pCHF3-Pro2Glu. For pCHF3-m2Glu- or pCHF3-m3P28-infiltrated regions, GFP mRNA accumulation was equivalent to wild-type pCHF3-Pro2Glu or pCHF3-P28, respectively (Figure 5D). These data indicate that two GW (G_5_W_6_ and G_116_W_117_) motifs of Pro2Glu are indispensable to suppression of local RNA silencing.

### Pro2Glu and P28 Enhance Accumulation of PVX RNA, and P28 Is a Putative Symptom Determinant

**Figure 6:**
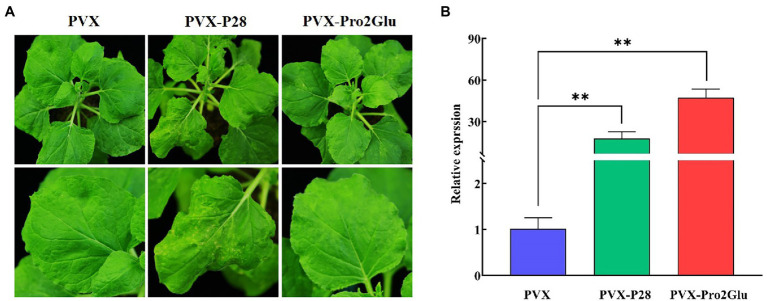
Pro2Glu and P28 enhanced the pathogenicity of potato virus X (PVX) in *N. benthamiana* plants. **(A)** The symptoms on *N. benthamiana* systemic leaves infiltrated by pGR106 empty vector (PVX), PVX-P28 or PVX-Pro2Glu at 22 dpi. **(B)** The relative expression of PVX in *N. benthamiana* systemic leaves at 22 dpi as determined by RT-qPCR. The error bars indicate standard error, *n* = 3. Differences between PVX and PVX-P28 or PVX-Pro2Glu are analyzed by one-way ANOVA and Tukey’s test (*p* < 0.05), ^**^p < 0.01.

Most VSRs have been suggested to enhance synergistically the severity of infection when they are expressed from a heterologous virus-based expression vector (Cao et al., 2005; Cañizares et al., 2008; Tatineni et al., 2012; Samuel et al., 2016). To test the hypothesis that Pro2Glu and P28 could enhance pathogenicity of a heterologous virus, we took advantage of the PVX-based heterologous gene expression system to express the Pro2Glu and P28 proteins in WT *N. benthamiana* plants. *Agrobacterium* cultures containing pGR106 empty vector (PVX) or recombinant PVX constructs expressing individual Pro2Glu and P28 were infiltrated in the third and fourth leaves from top of 4-week-old *N. benthamiana* plants. The inoculated plants were monitored for symptom development. At 10 dpi, non-inoculated systemic leaves exhibited severe mosaic symptoms in *N. benthamiana* plants inoculated with PVX and PVX-P28 (Supplementary Figure 5). By 22 dpi, the upper leaves inoculated with PVX-P28 appeared crinkled and had necrotic lesions (Figure 6A). However, the plants inoculated with PVX-Pro2Glu showed mild mosaic symptom at 10 dpi (Supplementary Figure 5). By 22 dpi, mild mosaic symptoms in the plants inoculated with PVX-Pro2Glu were similar to the control (PVX-inoculated) plants (Figure 6A). These observations indicate P28 is a putative symptom determinant, and Pro2Glu does not aggravate the symptoms of PVX.

To determine the genomic RNA copies of PVX, RT-qPCR was performed using total RNA extracted from systemically infected leaves. The result showed that PVX-Pro2Glu and PVX-P28 accumulated with, respectively, 47- and 18-fold increases compared to wild-type PVX at 22 dpi (Figure 6B), indicating that Pro2Glu and P28 significantly enhanced heterologous virus PVX accumulation in *N. benthamiana* plants.

## Discussion

First recognized as a distinct virus in 1946, SMoV is the most economically important virus infecting strawberries (*Fragaria* spp.) both in terms of its geographic distribution and potential yield losses it can cause (Prentice and Harris, 1946). Its severe strains may reduce vigor and yield of strawberry by up to 30%, and even 80% in mixed infections with strawberry vein banding virus (SVBV), strawberry crinkle virus, and/or strawberry mild yellow edge virus (Thompson et al., 2002; Thompson and Jelkmann, 2003; Fan et al., 2022). However, many attempts to characterize SMoV further had limited success because of difficulties in purifying the virus from infected plants and its distinct genomic organization. Hence, it remains unclear how SMoV induces disease and interacts and interferes with host components (Bhagwat et al., 2016; Mann et al., 2017, 2019). In the present study, we report that SMoV RNA2 genome encodes two suppressors of RNA silencing. SMoV Pro2Glu and P28 suppressed local and systemic silencing triggered by single- but not double-stranded GFP RNA. Both Pro2Glu and P28 were localized in the nucleus and cytoplasm. We present evidence that Pro2Glu ability to suppress host RNA silencing requires two conserved GW motifs, and deletion of 11 amino acids at the C-terminus destabilizes Pro2Glu protein. Finally, we showed that Pro2Glu and P28 enhanced PVX accumulation in *N. benthamiana* plants, and that P28 is a putative symptom determinant.

Several VSRs have been reported for viruses in *Secoviridae* family (Liu et al., 2004; Yaegashi et al., 2007; Karran and Sanfaçon, 2014; Kong et al., 2014; Stewart et al., 2017). However, our study identified for the first time the virus suppressor of RNA silencing encoded by a stramovirus. Pro2Glu, the first glutamic protease encoded by a positive-strand RNA virus, is unique to a few members of the family *Secoviridae* (Mann et al., 2019). It plays an essential role when SMoV polyprotein was processed into mature proteins (Mann et al., 2019). In our study, we found Pro2Glu acted as a suppressor to counter host RNA silencing. P28, located downstream of Pro2Glu, also suppressed host RNA silencing. Analyses of total RNA, siRNA and soluble proteins from agroinfiltrated leaf patches revealed that Pro2Glu possess a stronger ability to suppress host defense than P28; however, the level of suppression of RNA silencing by Pro2Glu and P28 was weak compared to that of strong VSR P19 of TBSV. This may be because P19 possesses very high affinity to duplexes 20–22 nt siRNA, at the same time, it induces the expression of host miR168 with the result that the formation of RISC is blocked (Danielson and Pezacki, 2013; Kontra et al., 2016).

Pro2Glu shares similarity with CPMV SCP (VSR of CPMV) in genome location (Liu et al., 2004), suggesting that Pro2Glu may suppress host defense in the same manner as SCP of CPMV. Unfortunately, the mechanism of suppression by CPMV SCP is still unclear. Previous studies have reported that the nuclear import was mandatory for some VSRs (Haas et al., 2008; Yang et al., 2011; Perez-Canamas and Hernandez, 2018). We explored the subcellular localization of Pro2Glu and P28 and found they were both localized in nucleus and cytoplasm. Many studies reported that the loss of NLS in VSR abolished the activity of the silencing suppressor, such as 2b of CMV, V2 of mulberry mosaic dwarf-associated virus, P6 of cauliflower mosaic virus (CaMV) and SVBV, and so on (Lucy et al., 2000; Wang et al., 2004; González et al., 2010; Feng et al., 2018; Yang et al., 2018). In the present study, we predicted that the GVSNRKKHRRG motif between amino acids 329 and 339 might be the NLS of the Pro2Glu protein. However, the subcellular localization of Glu^dm329-339aa^ mutant with the 329GVSNRKKHRRG339 motif deleted was still distributed in the nucleus, indicating that the motif may not be NLS of Pro2Glu. We need a more accurate tool to predict the NLS of Pro2Glu. However, deletion of the 329GVSNRKKHRRG339 motif destabilized Pro2Glu protein, thus the role of this motif in RNA silencing remained inconclusive.

Two conserved GW motifs of Pro2Glu are indispensable to its suppressor activity. The GW motif could “hook up” with AGOs to counteract host RNA silencing-based defenses (Lian et al., 2009; Danielson and Pezacki, 2013). Previous studies also reported that AGO1 localization in the cell appears to be in both cytoplasm and nucleus (González et al., 2010; Gupta et al., 2019). Therefore, we speculate that Pro2Glu protein may suppress the host RNA silencing by interacting directly with AGO1. VSRs may target multiple pathways in host cells, such as CMV 2b, CaMV P6, High Plains wheat mosaic virus (HPWMoV) P7, and so on (Zhang et al., 2006; Feng et al., 2018; Gupta et al., 2019). In our study, Pro2Glu and P28 both failed to suppress local silencing triggered by dsGFP RNA, demonstrating that they probably target the upstream steps of dsRNA production. It is possible that Pro2Glu and P28 directly interact with suppressor of gene silencing 3 (SGS3) or RNA-dependent RNA polymerase 6 (RDR6), or hinder their function, just like V2 protein of tomato yellow leaf curl virus and beet curly top virus (Glick et al., 2008; Fukunaga and Doudna, 2009; Luna et al., 2017). However, failing in detecting an interaction between Pro2Glu/P28 and SGS3/RDR6 in yeast (Supplementary Figure 6) suggests that Pro2Glu and P28 may hinder the function of SGS3 or RDR6 by other pathway. Further study is necessary to clarify the mechanism of Pro2Glu and P28.

VSRs generally are multifunctional. Many VSRs also play a pathogenicity-determining role. The most typical VSR is CMV 2b. Other recently identified VSRs include SVBV P6 (Feng et al., 2018), HPWMoV P7 and P8 (Gupta et al., 2018, 2019), apple geminivirus V2 (Zhan et al., 2018), raspberry bushy dwarf virus 1b (Masamichi et al., 2019), tomato leaf curl Palampur virus AC4 (Kulshreshtha et al., 2019) and so on, all aggravating the pathogenicity of heterologous virus PVX. In our study, we found that P28 exacerbated significantly the symptoms of PVX, indicating that P28 might play an important role in pathogenicity or virulence. Plant miRNAs regulate negatively the expression of mRNA, and involved in symptom development (Wang et al., 2012). Some VSRs could affect the miRNA-induced gene-silencing pathway (Tatineni et al., 2012; Kong et al., 2014). It is possible that P28 has an effect on the miRNA-induced gene-silencing pathway. In contrast, Pro2Glu with PVX caused inconspicuous symptoms compared to PVX, suggesting that Pro2Glu is likely to have little relevance to pathogenicity. Interestingly, RT-qPCR assay showed that Pro2Glu significantly enhanced the accumulation of PVX RNA at 22 dpi, demonstrating that Pro2Glu may help PVX infect plants *via* suppressing host defense responses involving RNA silencing, but may not influence the pathogenicity of PVX.

## Conclusion

This study increased understanding of the function of Pro2Glu and P28 proteins, which provided a starting point for clarifying the pathogenic mechanism of strawberry mottle virus. In addition, this study identified the stramovirus suppressor of RNA silencing for the first time, which provides a foundation for studying other viruses in the genus *Stramovirus*.

## Data Availability Statement

The original contributions presented in the study are included in the article/Supplementary Material, further inquiries can be directed to the corresponding author.

## Author Contributions

LF conducted experiments and wrote the manuscript. CH performed experiments and interpreted data. DG performed experiments and took photos. TX carried out experiments and cultivated experimental plants. FX contributed to experimental design. JY contributed to manuscript revision. BZ contributed to experimental design and data analysis. SL contributed to manuscript revision. HW contributed to experimental design and manuscript review and editing. All authors contributed to the article and approved the submitted version.

## Funding

The National Key R&D Program of China (2019YFD1001800) funded this study.

## Conflict of Interest

The authors declare that the research was conducted in the absence of any commercial or financial relationships that could be construed as a potential conflict of interest.

## Publisher’s Note

All claims expressed in this article are solely those of the authors and do not necessarily represent those of their affiliated organizations, or those of the publisher, the editors and the reviewers. Any product that may be evaluated in this article, or claim that may be made by its manufacturer, is not guaranteed or endorsed by the publisher.
