# Supplementary material for: Identification of Silencing Suppressor Protein Encoded by Strawberry Mottle Virus
Source: Front Plant Sci. 2022 May 31;13:786489. doi: 10.3389/fpls.2022.786489 (PMC9195133; doi:10.3389/fpls.2022.786489)
Supplement: Supplementary file 1 [file Data_Sheet_1.pdf]

frontiers

Supplementary Material

Supplementary Table 1. Primers used in this study (restriction sites were underlined)

Primer	Nucleotide sequence (5´-3´)
X1(KpnI)-F	GGTACCATGAGTTACCTCTGCGATGACGT
X1(XbaI)-R	TCTAGATCACTGTTCAACACATTGTGCTACCGT
X2(KpnI)-F	GGTACCGGTGGCATCTCTGTTGGTG
X2(XbaI)-R	TCTAGATCATTGCTCGTAAGCGGGACAGT
Hel(BamHI)-F	GGATCCGGGGACAAGATACAAACTCAC
Hel(PstI)-R	CTGCAGTCATTGTTCGGTAGCAACTTCAAT
Vpg(BamHI)-F	GGATCCGGAGGGTATGCCGAATCG
Vpg(PstI)-R	CTGCAGTCATTGTTCATATGCACGCACAC
Pro(BamHI)-F	GGATCCGGTGCCTCCGACTCCCAG
Pro(PstI)-R	CTGCAGTCATTGCTGAACAGCCACCTCAG
RdRp(BamHI)-F	GGATCCGGCATGGACATTGATATCTTGG
RdRp(PstI)-R	CTGCAGTCAACCAAGAACGGCGGT
MP(KpnI)-F	GGTACCATGTTTTTGTTTGTGTTGTACG
MP(XbaI)-R	TCTAGATCATTCTTCATAAGCTCGGGT
CP(BamHI)-F	GGATCCGGCTTGCCTGAAGATGTCAC
CP(PstI)-R	CTGCAGTCAGGGTTTCACATCTAGAACAG

Supplementary Table 1 (continued)

Primer	Nucleotide sequence (5´-3´)
Pro2Glu (BamHI/SmaI/ClaI)-F	GGATCC/CCCGGG/ATCGATGCCTTTCCTTTTGGATG
Pro2Glu (PstI/KpnI/SalI)-R	CTGCAG/GGTACC/GTCGACTCAGGGTTTAGGTCTACCTCT
P28 (BamHI/ApaI/ClaI)-F	GGATCC/CCCGGG/ATCGATAAGTACCCCGAAGGTGAGCT
P28 (PstI/KpnI/SalI)-R	CTGCAG/GGTACC/GTCGACTCACTGACCGAGGGCACCG
$Glu^{dm329-339aa}$ (BamHI/Sma I)-F	GGATCC/CCCGGGGCCTTTCCTTTTGGATG
$Glu^{dm329-339aa}$ (Pst I/Kpn I)-R	CTGCAG/GGTACCTCAGGGTTTAGGTCTAACTCTGTT
G-35	ATGGATATTCTCATCATTAG
G-36	CTATGTCCCTGCGCGGACATATG
PP2A-F	GTGAAGCTGTAGGGCCTGAGC
PP2A-R	CATAGGCAGGCACCAAATCC

Supplementary Table 2. The number of *N. benthamiana* 16c plants systemically silencing at 28 dpi.

Construct (35S-GFP+)	No. plants infiltrated	No. plants systemically silenced	Suppression efficiency (%)
Vector	20	19	5.0
X1	12	12	0
X2	12	12	0
Hel	12	12	0
Vpg	12	12	0
Pro	12	12	0
RdRp	12	11	8.3
MP	12	12	0
CP	12	11	8.3
Pro2Glu	34	11	67.6
P28	34	12	64.7
P19	20	2	90.0

Note: The plants with major and minor veins of upper young leaves turning red were assumed to be silenced systemically.

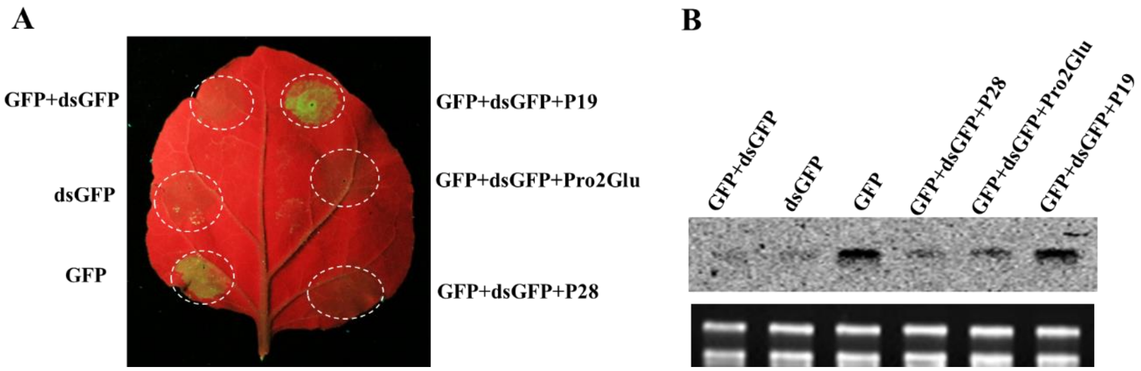

Supplementary Figure 1. Pro2Glu and P28 failed to suppress local silencing triggered by dsGFP. **(A)** The observation of GFP fluorescence in wild-type *N. benthamiana* leaf infiltrated by 35S-GFP, 35S-dsGFP, 35S-GFP+35S-dsGFP, 35S-GFP+35S-dsGFP plus pCHF3-Pro2Glu, -P28 or -P19 at 3 dpi. **(B)** The analysis of GFP mRNA in infiltrated leaf patches by northern blot at 3 dpi. DIG-labeled *GFP*-specific probe was used to detect the GFP mRNA. GelStain staining was used to visualize the loading controls for the mRNA.

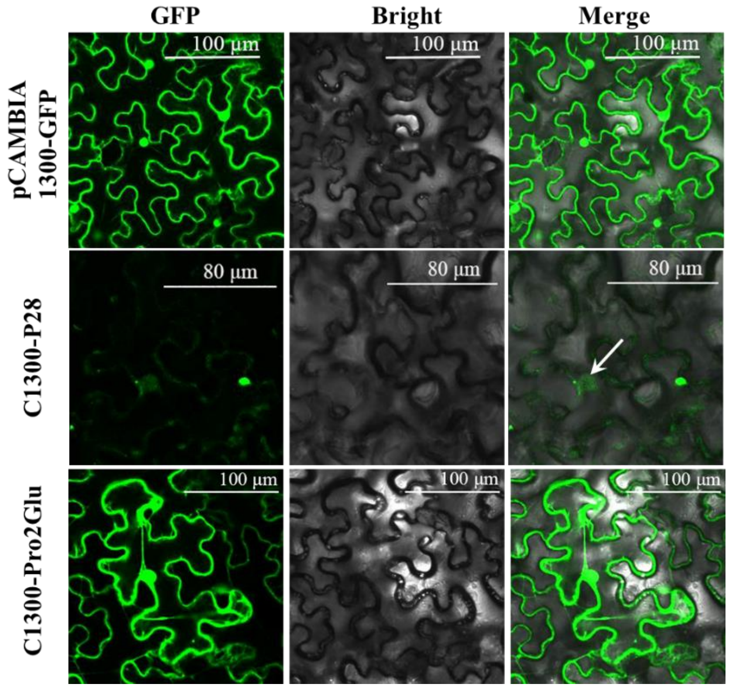

Supplementary Figure 2. The subcellular localization of Pro2Glu and P28. Both Pro2Glu and P28 were localized in nucleus and cytoplasm.

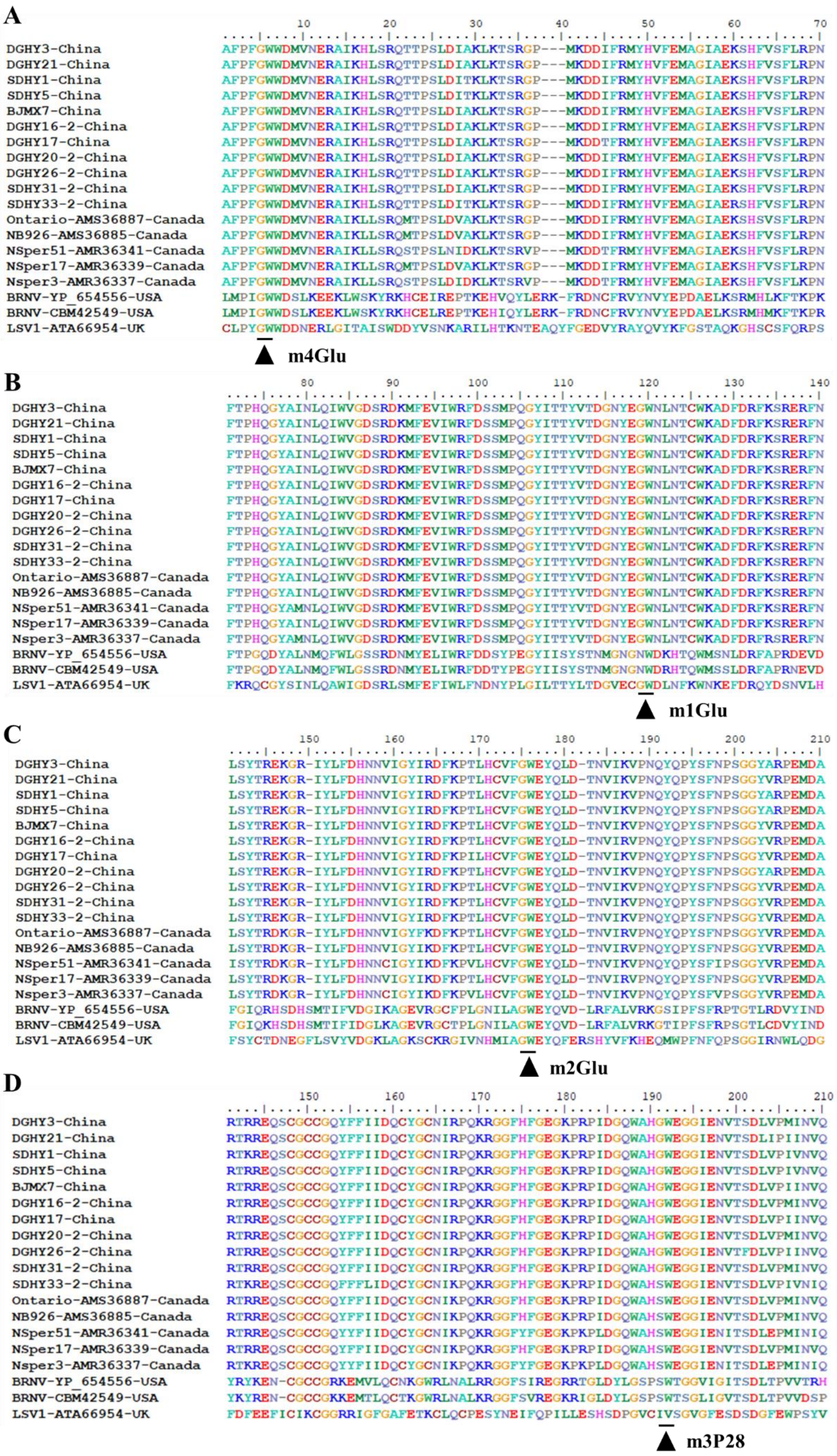
A
m4Glu
B
m1Glu
C
m2Glu
D
m3P28

Supplementary Figure 3. Sequence alignments of the putative Pro2Glu (A, B and C) and P28 (D) domain for selected members of the family *Secoviridae*. The deduced amino acid sequences of the Pro2Glu and P28 proteins from 11 SMoV Chinese isolates (Fan et al., 2021), five SMoV Canadian isolates (Bhagwat et al., 2016), two BRNV isolates (Mann et al., 2018) and one LSV1 isolate (Mann et al., 2018) were used for the alignments. The conserved GW motifs were found in Pro2Glu and P28. **(A)** The G_5W_6 motif was conserved in Pro2Glu. **(B)** The $G_{116}W_{117}$ motif was conserved in SMoV Pro2Glu. **(C)** The $G_{171}W_{172}$ motif was conserved in Pro2Glu. **(D)** The $G_{190}W_{191}$ motif was conserved in P28 of the Chinese isolates of SMoV. (Accession numbers: DGHY3, DGHY21, SDHY1, SDHY5, and BJMX7 is QPM65692−QPM65696, respectively; DGHY16-2, DGHY17, DGHY20-2, DGHY26-2, SDHY31-2, and SDHY33-2 is QRR19197−QRR19202, respectively; Ontario is AMS36887, NB926 is AMS36885, NSper51 is AMR36341, NSper17 is AMR36339, NSper3 is AMR36337, BRNV-YP_654556-USA is YP_654556, BRNV-CBM42549-USA is CBM42549, and LSV1 is ATA66954, respectively.)

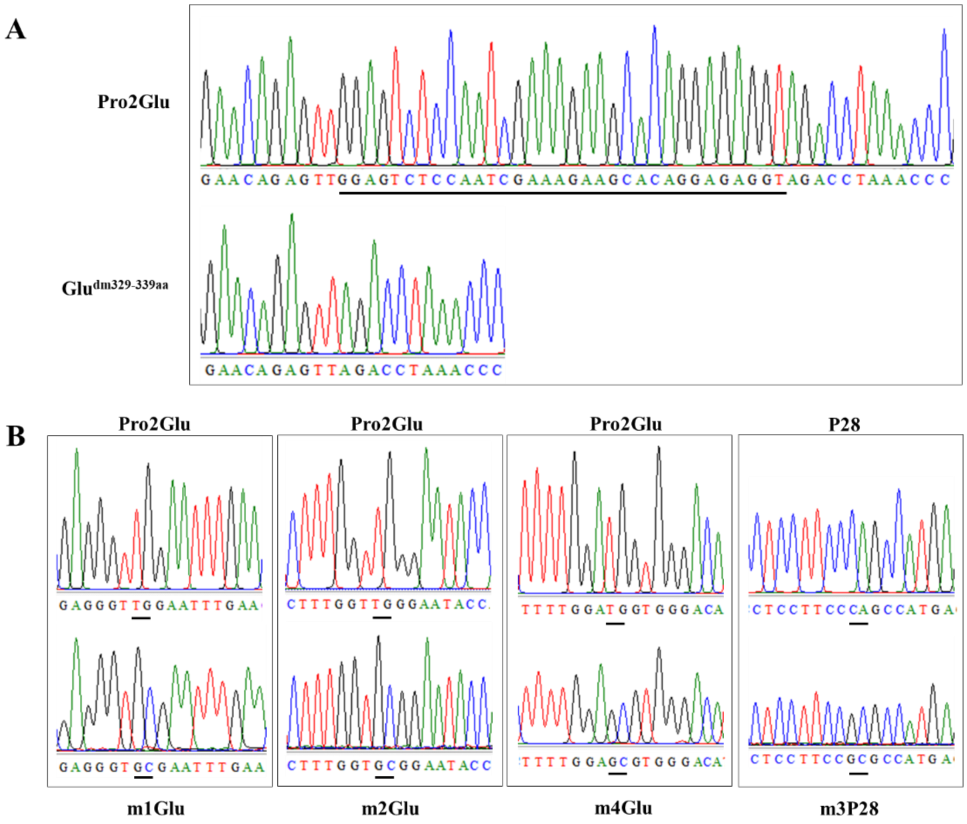

Supplementary Figure 4. The sequencing results of Pro2Glu and P28 mutants. **(A)** The 329GVSNRKKHRRG339 coding region of Pro2Glu was deleted successfully. **(B)** The GW motifs of Pro2Glu and P28 were disrupted successfully through W_6A (m4Glu), $W_{117}A$ (m1Glu), $W_{172}A$ (m2Glu), and $W_{191}A$ (m3P28) site-directed mutagenesis. Forward sequencing results for Pro2Glu and its mutants, reverse sequencing results for P28 and its mutant, and deleted or mutated bases were underlined.

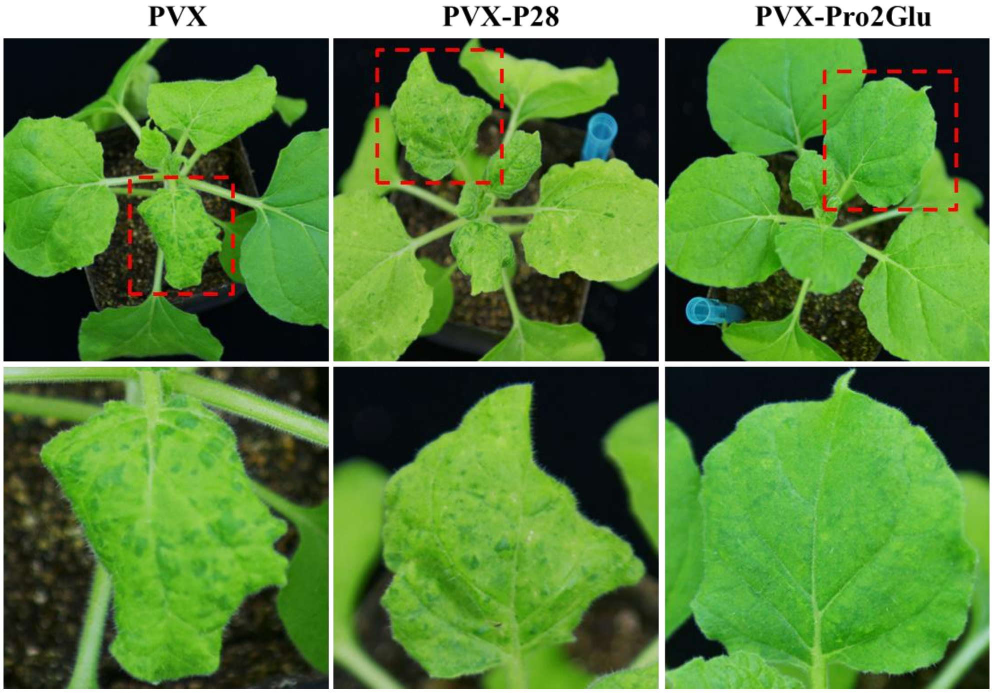

Supplementary Figure 5. The symptoms on *N. benthamiana* systemic leaves infiltrated by pGR106 empty vector (PVX), PVX-P28 or PVX-Pro2Glu at 10 dpi.

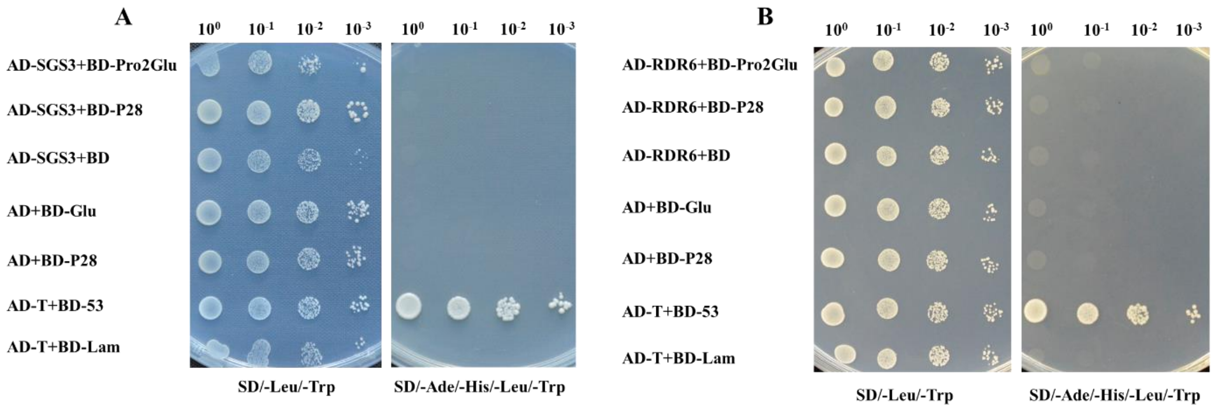

Supplementary Figure 6. Analysis of the interaction between Pro2Glu/P28 and SGS3/RDR6 by Y2H. **(A)** Interactions between Pro2Glu/P28 and SGS3 in yeast. **(B)** Interactions between Pro2Glu/P28 and RDR6 in yeast. pGADT7 (AD)-SGS3 or -RDR6 and pGBKT7 (BD)-Pro2Glu or -P28 were co-transformed into yeast cells and selected on synthetic dextrose (SD) lacking -Leu/-Trp or -Ade/-His/-Leu/-Trp medium in 10-fold serial dilutions for 72 h. Yeasts co-transformed with AD-T plus BD-53 or BD-Lam were designated as positive or negative controls, respectively.
